# Shengjiang San suppresses C5a/C5aR1-mediated neutrophil activation and NET formation to alleviate acute lung injury

**DOI:** 10.3389/fphar.2026.1955412

**Published:** 2026-10-21

**Authors:** Dan Zhang, Xinxin Li, Jia Zeng, Ding Cheng, Yuze Wang, Shuqi Liu, Beibei Shao, Chengfeng Liu, Haiming Chen, Yicheng Liu, Xin Zhao, Guangrui Huang, Anlong Xu

**Affiliations:** 1 School of Life Sciences, Beijing University of Chinese Medicine, Beijing, China; 2 State Key Laboratory of Traditional Chinese Medicine Syndrome, Guangzhou, China; 3 Beijing Research Institute of Chinese Medicine, Beijing University of Chinese Medicine, Beijing, China; 4 Institute of Basic Theory of Traditional Chinese Medicine, China Academy of Chinese Medical Sciences, Beijing, China; 5 School of Chinese Materia Medicine, Beijing University of Chinese Medicine, Beijing, China; 6 State Key Laboratory of National Security Specially Needed Medicines, Beijing, China; 7 Department of Emergency, Guang’anmen Hospital, Beijing, China; 8 Wudang Medical Research Institute, Beijing University of Chinese Medicine, Beijing, China; 9 Hong Kong Institute of Advanced Studies, Sun Yat-sen University, Hong Kong, China

**Keywords:** acute lung injury, Shengjiang San, C5a/C5aR1, neutrophil, neutrophil extracellular traps, ferulic acid

## Abstract

**Background:**

Excessive neutrophil activation and neutrophil extracellular trap (NET) formation contribute to hyperinflammation and disruption of the alveolar capillary barrier in acute lung injury (ALI). C5a/C5aR1 signaling links complement activation to neutrophil migration, Ca^2+^ mobilization, ROS production, and NET formation. This study investigated whether Shengjiang San (SJS) regulates these responses in ALI and explored candidate circulating constituents.

**Methods:**

ALI was induced in mice by intratracheal LPS administration, and SJS treatment was initiated before LPS exposure. Lung injury, barrier integrity, neutrophil responses, NET formation, C5a/C5aR1 changes, and lung transcriptomic profiles were assessed. Purified murine neutrophils were stimulated with C5a or PMA to evaluate the effects of SJS-containing serum. Serum pharmacochemistry, plasma exposure analysis, biolayer interferometry, molecular docking, and neutrophil assays were used to characterize ferulic acid.

**Results:**

SJS reduced pulmonary inflammation, edema, vascular leakage, neutrophil accumulation and activation, and NET formation, while preserving the integrity of the alveolar capillary barrier. Transcriptomic profiling showed enrichment of complement activation, neutrophil activation, and NET formation among genes differentially expressed between the SJS and Model groups. SJS also lowered C5a concentrations in plasma and lung homogenates and reduced pulmonary C5aR1 expression. In neutrophils, SJS-containing serum significantly suppressed C5a-stimulated neutrophil functions, including migration, Ca^2+^ flux, oxidative burst, and NET release, while PMX205 produced a similar inhibitory pattern. SJS-containing serum also inhibited PMA-induced NET formation. Ferulic acid was detected in serum as a prototype constituent with measurable plasma exposure, showed concentration-dependent binding to recombinant human C5a with an apparent *K*_*D*_ of 21.65 μM, and reduced histone H3 citrullination and NET formation in C5a-stimulated neutrophils.

**Conclusion:**

SJS attenuated LPS-induced ALI and restrained neutrophil activation and NET formation mediated by C5a/C5aR1 signaling. Ferulic acid was identified as a candidate circulating constituent that reduced C5a-activated NET formation.

## 1 Introduction

Acute lung injury (ALI) and its more severe form, acute respiratory distress syndrome (ARDS), are life threatening syndromes characterized by diffuse pulmonary inflammation and disruption of the alveolar-capillary barrier (Bos and Ware, 2022). Despite advances in supportive care, ALI/ARDS remains associated with high mortality, particularly in severe cases (Bellani et al., 2016). In a multicenter prospective study in mainland China, hospital mortality reached 46.3% overall and 56.2% among patients with severe ARDS (Huang et al., 2020). These data highlight the imperative to elucidate the pathological mechanisms underpinning sustained lung injury and to develop targeted pharmacological therapies. A hallmark of ALI is not merely a robust inflammatory response but a failure to effectively constrain innate immune effector programs. Neutrophils are essential components of host defense, but their functions vary according to their activation states and tissue environment (Zhang et al., 2024). In ALI, the persistent accumulation of pro-inflammatory neutrophils serves to exacerbate pulmonary inflammation and tissue injury (Wang et al., 2022). In contrast, neutrophil populations that emerge during the resolution phase may promote inflammation resolution and epithelial repair (Tavares et al., 2025). Therefore, therapeutic strategies should selectively restrain pathological neutrophil activation and NET formation rather than broadly suppress neutrophil function.

Neutrophil extracellular traps (NETs) are extracellular chromatin structures decorated with histones and neutrophil granule proteins, including myeloperoxidase (MPO) and neutrophil elastase (NE) (Wang et al., 2024). Although NET formation contributes to antimicrobial defense, excessive or poorly cleared NETs can injure host tissues. Clinical studies have demonstrated that NETs accumulate abnormally in both the circulation and alveolar space of patients with ALI/ARDS, correlating with disease severity and adverse outcomes (Lefrançais et al., 2018; Bendib et al., 2019; Aoki et al., 2025). Mechanistically, excessive NETs inflict direct injury on alveolar epithelial and pulmonary endothelial cells *via* released cytotoxic cargo, including histones and MPO, while concurrently disrupting endothelial integrity (Saffarzadeh et al., 2012; Sha et al., 2024). Furthermore, NETs amplify localized inflammatory and procoagulant responses, thereby compromising alveolar–capillary barrier integrity and exacerbating lung injury (Zhu et al., 2023; Caudrillier et al., 2012). The pathogenic role of NETs is further supported by experimental intervention studies. Disruption of upstream signaling events that promote NET formation has been shown to reduce pulmonary NET accumulation, inflammation, microvascular thrombosis, and tissue injury in models of ALI and sepsis (Burkard et al., 2023; Alsabani et al., 2022). Consistent with this concept, a lung-targeted formulation designed to inhibit NET formation and promote the degradation of released NETs reduced pulmonary NET accumulation and was associated with improved lung pathology, respiratory function, and survival in experimental ALI (Kim et al., 2025). The available evidence positions excessive NET formation as a potentially tractable component of ALI/ARDS pathogenesis and draws attention to the upstream inflammatory signals that initiate and sustain NET release within the injured lung.

Identifying the upstream signals that promote pathological neutrophil activation and excessive NET formation is essential for developing targeted therapies for ALI. Recent intervention studies have shown that selective blockade of NET promoting inflammatory signals can reduce NET accumulation and ameliorate experimental lung injury (Burkard et al., 2023; Alsabani et al., 2022). Among these signals, C5a, a potent anaphylatoxin generated during complement activation, links complement dysregulation to neutrophil mediated tissue injury. By binding to C5aR1 on neutrophils, C5a promotes β2 integrin dependent adhesion, recruitment, and cellular activation (Miyabe et al., 2017; Seiler et al., 2023). C5aR1 signaling also triggers intracellular Ca^2+^ mobilization and primes neutrophils for oxidative burst and granule release (Schreiber et al., 2009). In an inflammatory environment, these responses can progress to ROS dependent NET formation, shifting neutrophil activity from antimicrobial defense toward the amplification of inflammation and tissue injury (Chen et al., 2022). This process is particularly harmful in the lung, where NET-associated histones and granule proteins can directly injure alveolar epithelial and vascular endothelial cells and aggravate lung injury (Saffarzadeh et al., 2012). Experimental studies further implicate complement signaling in neutrophil-mediated pulmonary injury. Complement depletion reduced NET release, pulmonary vascular permeability, edema, and mortality in antibody-induced ALI (Cleary et al., 2020). More specifically, neutralization of C5a reduced pulmonary neutrophil accumulation and vascular permeability, whereas disruption of C5a receptor signaling decreased extracellular histone accumulation and alveolar permeability in experimental lung injury (Mulligan et al., 1996; Bosmann et al., 2013). More direct evidence linked C5aR1 signaling to pulmonary NET formation, as C5aR1 inhibition reduced NET accumulation, lung inflammation, and tissue injury (Silva et al., 2023). However, whether SJS can limit neutrophil activation and excessive NET formation associated with C5a/C5aR1 signaling while preserving the integrity of the alveolar capillary barrier in ALI remains unclear.

Traditional Chinese medicine (TCM), characterized by multicomponent and multitarget actions, may offer therapeutic candidates for complex inflammatory disorders. Shengjiang San (SJS) is a classical Chinese medicine formula traditionally prescribed for febrile disorders (Zhang et al., 2018). In murine sepsis, SJS improved survival and reduced lung injury, accompanied by temporal regulation of the inflammatory response and inhibition of macrophage pyroptosis through the NF-κB and NLRP3 pathway (Yan et al., 2023). Protective effects have also been reported in sepsis induced intestinal and myocardial injury. In these models, SJS preserved intestinal epithelial barrier integrity, regulated microbial metabolism and macrophage polarization, and suppressed p38 MAPK activation (Yan et al., 2026; Qian et al., 2019). These studies establish an experimental basis for investigating SJS in inflammatory organ injury, but the mechanisms examined to date have focused mainly on macrophages, inflammatory cytokines, and intestinal immune regulation. In particular, it is unclear whether SJS regulates C5a/C5aR1 signaling, pathological neutrophil activation, and excessive NET formation, or whether such regulation contributes to protection of the alveolar epithelial and pulmonary vascular endothelial barriers. Chemical profiling and pharmacokinetic studies have identified numerous constituents of SJS (Yan et al., 2026; Zhu et al., 2017), but the circulating constituents involved in the regulation of this neutrophil response have not been defined. We therefore investigated whether SJS attenuates ALI in association with regulation of C5a/C5aR1-associated neutrophil activation and NET formation and sought to identify circulating constituents that may contribute to these protective effects.

Accordingly, we hypothesized that SJS alleviates ALI by restraining C5a/C5aR1 related neutrophil activation and excessive NET formation, thereby preserving pulmonary barrier integrity. To test this hypothesis, we combined an LPS-induced mouse model of ALI with lung transcriptomic analysis and functional studies in primary neutrophils to examine the effects of SJS on complement activation, neutrophil responses, NET formation, and barrier injury. We also characterized the chemical composition of SJS and its serum exposed constituents, followed by binding and functional validation of candidate compounds. This integrated approach was used to investigate both the immunological mechanism and the circulating material basis underlying the protective effect of SJS in ALI.

## 2 Materials and methods

### 2.1 Reagents and antibodies

Lipopolysaccharide (LPS, *Escherichia coli* O55:B5) was purchased from Sigma-Aldrich (St. Louis, MO, USA). Recombinant mouse C5a and PMX205 were obtained from MCE (Shanghai, China). ELISA kits and fluorescent probes were obtained from commercial sources. Detailed information on reagents and antibodies, including catalog numbers, clones, and working dilutions, is provided in the Supplementary Material.

### 2.2 Preparation of SJS

SJS consisted of *Rheum palmatum* L. (WFO-0000404565), *Curcuma longa* L. (WFO-0000368193), *Bombyx mori* L., and *Cryptotympana atrata* (Fabricius) in a ratio of 4:3:2:1. Detailed information is provided in the Supplementary Material. All materials were purchased from Guang’anmen Hospital, China Academy of Chinese Medical Sciences, and authenticated by Prof. Guangrui Huang (Beijing University of Chinese Medicine, Beijing, China). The mixed herbs were refluxed twice with distilled water at 1:10 (w/v) for 1 h and 1:5 (w/v) for 1 h. The combined extracts were concentrated under reduced pressure at 70 °C to 0.6 g crude herb/mL and stored at −80 °C until use. For chemical analysis, the aqueous extract was lyophilized before UHPLC-Q-Orbitrap HRMS analysis.

### 2.3 Preparation of serum containing SJS

Serum containing SJS was prepared using Sprague-Dawley rats (n = 10 per group). Rats received SJS (7 g/kg/day) or distilled water by gavage for 7 days. Blood was collected from the abdominal aorta 2 h after the final administration. Serum was separated by centrifugation, filtered through a 0.22 μm membrane, heat-inactivated at 56 °C for 30 min, and stored at −80 °C until use.

### 2.4 Animals and ethics statement

Male C57BL/6J mice (6–8 weeks old, 20–22 g) were purchased from Beijing Vital River Laboratory Animal Technology Co., Ltd. (Beijing, China). Mice were housed under specific pathogen-free conditions at 22 °C ± 2 °C on a 12 h light/dark cycle with free access to food and water. All procedures were approved by the Animal Care and Use Committee of Beijing University of Chinese Medicine (No. BUCM-2023091003-3242) and complied with the National Institutes of Health Guide for the Care and Use of Laboratory Animals.

### 2.5 LPS-induced ALI model and drug administration

Mice were randomly assigned to six groups: Control, Model, SJS-L (1.125 g/kg), SJS-M (2.25 g/kg), SJS-H (4.5 g/kg), and DEX (5 mg/kg) (n = 6 per group). SJS or DEX was administered by gavage once daily for five consecutive days. Two hours after the final pretreatment, acute lung injury was induced by intratracheal instillation of LPS (5 mg/kg in PBS). Control mice received PBS alone. Drug administration was continued once daily after LPS challenge. Samples were collected 48 h after LPS instillation.

### 2.6 Histology and BALF analysis

Lung tissues were fixed in 4% paraformaldehyde, embedded in paraffin, sectioned at 5 μm, and stained with hematoxylin and eosin. Lung injury was scored according to the American Thoracic Society workshop report (Matute-Bello et al., 2011). The scoring criteria are shown in Supplementary Table S1.

BALF was collected through the trachea using three washes with Pre-cooled PBS. Total cell number and neutrophil number were determined from the cell pellet. Total protein concentration in BALF supernatants was measured using a BCA assay. IL-1β, TNF-α, and IL-6 levels were measured with commercial ELISA kits according to the manufacturers’ instructions.

### 2.7 Assessment of pulmonary barrier function

Pulmonary vascular permeability was assessed by Evans blue extravasation. Mice received 0.5% Evans blue (2 mL/kg, i.v.) 1 h before sacrifice. Lungs were collected, weighed, homogenized, and incubated in formamide at 37 °C for 24 h. Absorbance was measured at 630 nm, and Evans blue content was normalized to lung wet weight. Pulmonary edema was assessed by the wet-to-dry weight ratio. ZO-1, Occludin, and VE-cadherin were analyzed in lung sections by multiplex immunofluorescence with tyramide signal amplification. Images were acquired with a confocal microscope (AXR NSPARC, Nikon), and fluorescence signals were quantified using Image-Pro Plus software. For image presentation, identical lookup tables (LUTs) and intensity display ranges were applied across all experimental groups within each fluorescence channel.

### 2.8 RNA sequencing

Total RNA was extracted from lung tissue using TRIzol reagent (Invitrogen). RNA quality was assessed with an Agilent 2100 Bioanalyzer. Libraries were prepared from 1 μg total RNA and sequenced on an Illumina platform by LC Biotech (Hangzhou, China). Clean reads were aligned to the *Mus musculus* reference genome (GRCm38) using HISAT2. Transcript abundance was quantified with StringTie, and differential expression analysis was performed with edgeR. Differentially expressed genes were defined as those with | log2(fold change) | ≥ 1 and *P* < 0.05. GO and KEGG enrichment analyses were then performed. The RNA-sequencing data generated in this study have been deposited in the NCBI Gene Expression Omnibus (GEO) under accession number GSE345908.

### 2.9 Flow cytometric analysis of pulmonary neutrophils

Lung tissues were digested in RPMI-1640 containing collagenase IV (2 mg/mL) and DNase I (40 μg/mL) at 37 °C for 30 min. Cell suspensions were filtered, erythrocytes were lysed, and cells were blocked with anti-CD16/32. Cells were stained with antibodies against CD45, Ly6G, and CD11b for 30 min at 4 °C and analyzed on a CytoFLEX flow cytometer (Beckman Coulter). Neutrophils were identified by flow cytometry using sequential gating based on FSC-A/SSC-A, FSC-A/FSC-H singlet selection, exclusion of Zombie-positive cells, and final identification of CD11b^+^Ly6G^+^ cells within the viable CD45^+^ population.

### 2.10 Immunohistochemistry and immunoblot analysis

For immunohistochemistry, paraffin sections were incubated with primary antibodies against MPO, NE, and C5aR1, followed by HRP-DAB detection and hematoxylin counterstaining. Slides were scanned using a Leica Aperio VERSA 8 system, and integrated optical density was quantified in five randomly selected fields.

For immunoblotting, lung tissues were lysed in RIPA buffer containing protease and phosphatase inhibitors. Equal amounts of protein were separated by SDS-PAGE, transferred to PVDF membranes, and incubated with antibodies against CD11b, PAD4, Cit-H3, and β-actin. Signals were detected with ECL reagent and quantified with ImageJ.

### 2.11 Isolation and treatment of primary neutrophils

Primary neutrophils were isolated from mouse bone marrow or peritoneal lavage fluid, depending on the experimental design. Cells were purified using a discontinuous Percoll density gradient (70% and 55%). Cell purity exceeded 90% based on Ly6G staining by flow cytometry, and viability exceeded 95% as determined by trypan blue exclusion.

### 2.12 Transwell migration assay

This experiment used neutrophils isolated from mouse bone marrow. Neutrophil migration was assessed using 12-well Transwell inserts (3.0 μm pore size, Corning, NY, USA). Hoechst 33342-labeled neutrophils were seeded into the upper chamber at 2 × 10^5^ cells/well, and the lower chamber contained rmC5a or vehicle. After 1 h at 37 °C, cells on the lower surface of the membrane were fixed, stained with crystal violet, and quantified in five randomly selected fields per well using ImageJ. Cells in the lower chamber were imaged by fluorescence microscopy, collected, centrifuged, resuspended in 100 μL medium, and counted using a cell counter.

### 2.13 Immunofluorescence analysis of C5a-induced NET formation

Peritoneal neutrophils isolated from mice were used in this experiment. Neutrophils were seeded on poly-L-lysine-coated coverslips and pretreated with 10% serum containing SJS, PMX205, or vehicle for 1 h, followed by stimulation with rmC5a for 4 h. Cells were then fixed, permeabilized, and incubated with antibodies against MPO and Cit-H3, followed by Alexa Fluor-conjugated secondary antibodies. Nuclei were stained with DAPI, and extracellular DNA was stained with SYTOX Green. Images were acquired with an AXR NSPARC confocal microscope (Nikon). NET formation was assessed by MPO and Cit-H3 colocalization together with extracellular DNA staining.

### 2.14 Immunofluorescence and scanning electron microscopy analysis of PMA induced NET formation

Neutrophils were seeded on poly-L-lysine-coated coverslips and pretreated with serum containing SJS, GSK484, or vehicle, followed by stimulation with PMA for 4 h. Immunofluorescence staining for MPO, Cit-H3, DAPI, and SYTOX Green was performed as described above. For scanning electron microscopy, cells were fixed and processed using standard procedures for ultrastructural observation, and images were acquired to evaluate extracellular web-like structures.

### 2.15 Measurement of intracellular Ca^2+^ mobilization and ROS generation

Neutrophils were pretreated with 10% serum containing SJS, PMX205 (100 nM), or vehicle for 1 h. For intracellular Ca^2+^ measurement, cells were loaded with Fluo-4 AM (2 μM) for 30 min at 37 °C, washed, and stimulated with rmC5a. Fluo-4 fluorescence was measured immediately on a CytoFLEX flow cytometer. For intracellular ROS measurement, cells were stimulated with rmC5a for 1h and then incubated with DCFH-DA for 30 min at 37 °C. After washing, DCF fluorescence was measured on the same instrument. Data were analyzed with FlowJo.

### 2.16 Chemical characterization by UHPLC-Q-Orbitrap HRMS

The lyophilized aqueous extract of SJS and serum samples were analyzed by UHPLC-Q-Orbitrap HRMS. Compounds were identified or tentatively characterized by comparison with reference databases and MS/MS fragmentation patterns. Detailed chromatographic and mass spectrometric conditions are provided in the Supplementary Material.

### 2.17 Molecular docking

Molecular docking was performed to explore the potential binding mode between ferulic acid and C5a. The protein structure was obtained from the Protein Data Bank, and docking was conducted using AutoDock Tools and AutoDock Vina. The selected binding pose was visualized using PyMOL and BIOVIA Discovery Studio Visualizer.

### 2.18 Statistical analysis

Investigators were blinded to group allocation during histological scoring and image quantification. Data were analyzed using GraphPad Prism 10.3.0 (GraphPad Software, CA, USA). Results are presented as mean ± SEM. Normality was assessed by the Shapiro-Wilk test. For comparisons among multiple groups, ordinary one-way ANOVA followed by Dunnett’s *post hoc* test was used when variances were equal. When variance heterogeneity was detected by the Brown-Forsythe test (*P* < 0.05), Welch’s ANOVA followed by Dunnett’s T3 multiple comparisons test was applied. *P* < 0.05 was considered statistically significant.

## 3 Results

### 3.1 SJS attenuated LPS-induced lung injury and inflammatory responses

The protective effects of SJS were first evaluated in an LPS-induced mouse model of ALI (Figure 1A). LPS challenge induced inflammatory cell infiltration, alveolar wall thickening, and interstitial edema. These histopathological abnormalities were attenuated by SJS pretreatment, as evidenced by lower lung injury scores and reduced alveolar wall thickness relative to the model group (Figures 1B–D). Consistent with the histological findings, SJS pretreatment reduced the LPS-induced increases in total inflammatory cells and neutrophils in BALF (Figures 1E,F) and decreased the proportion of neutrophils in peripheral blood (Supplementary Figure S1A). SJS pretreatment also reduced the LPS-induced elevations in IL-1β, TNF-α, and IL-6 levels in BALF and serum (Figures 1G–L). In lung tissue, SJS reduced *Il1b*, *Tnf*, and *Il6* mRNA expression and decreased immunohistochemical staining for the corresponding proteins (Supplementary Figure S1B-H). These results show that SJS pretreatment attenuated pulmonary inflammation and histological injury in LPS-induced ALI.

**FIGURE 1:**
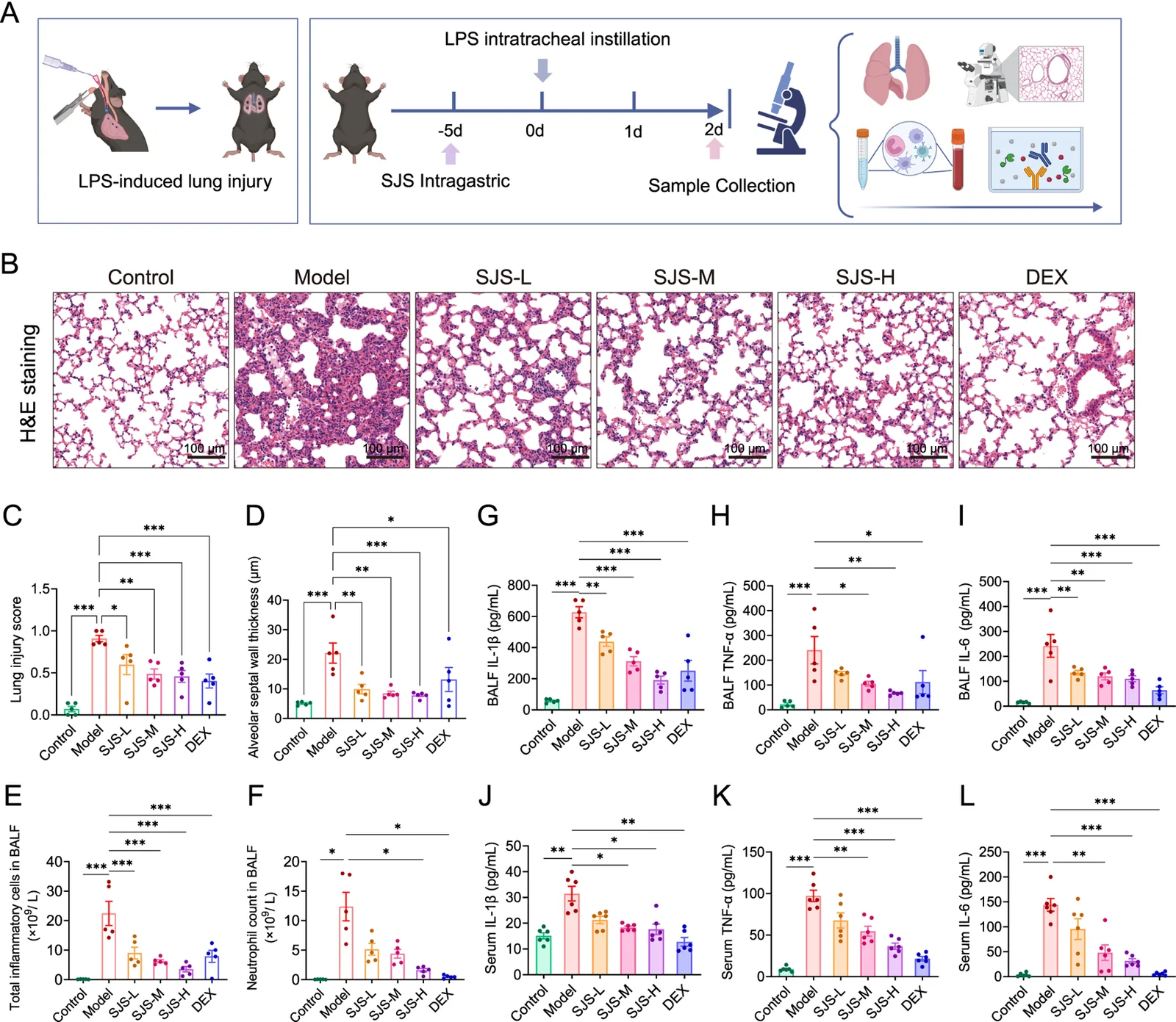
SJS attenuates LPS-induced lung histopathological damage and inflammatory responses in mice. **(A)** Schematic illustration of the experimental design. **(B)** H&E staining revealing the histology (scale bar = 100 μm). **(C)** Lung injury score. **(D)** Mean alveolar wall thickness. **(E)** Total inflammatory cell counts in BALF. **(F)** Neutrophil counts in BALF. **(G–I)** Levels of IL-1β, TNF-α, and IL-6 in BALF. **(J–L)** Serum levels of IL-1β, TNF-α, and IL-6. Data are presented as mean ± SEM (C-I, n = 5; J-L, n = 6). ^*^ *P* < 0.05, ^**^ *P* < 0.01, ^***^ *P* < 0.001 vs. Model. Created in BioRender.com ZHANG, D. (2026) https://BioRender.com/bntu044.

### 3.2 SJS preserved alveolar-capillary barrier integrity

To determine whether SJS protected the alveolar-capillary barrier, pulmonary vascular leakage and barrier integrity were assessed. LPS challenge increased Evans blue extravasation into lung tissue, whereas SJS pretreatment reduced dye leakage (Figures 2A,B). SJS pretreatment also reduced the LPS-induced increases in the lung wet-to-dry ratio and BALF total protein concentration (Figures 2C,D), indicating reduced pulmonary edema and alveolar-capillary permeability. Immunofluorescence staining showed that LPS challenge reduced the fluorescence intensity and disrupted the continuous distribution of ZO-1, Occludin, and VE-cadherin in lung tissue. SJS pretreatment preserved the intensity and continuous distribution of these junctional proteins, as confirmed by quantitative analysis (Figures 2E–H). These results show that SJS pretreatment reduced pulmonary vascular leakage and preserved alveolar-capillary barrier integrity in LPS-induced ALI.

**FIGURE 2:**
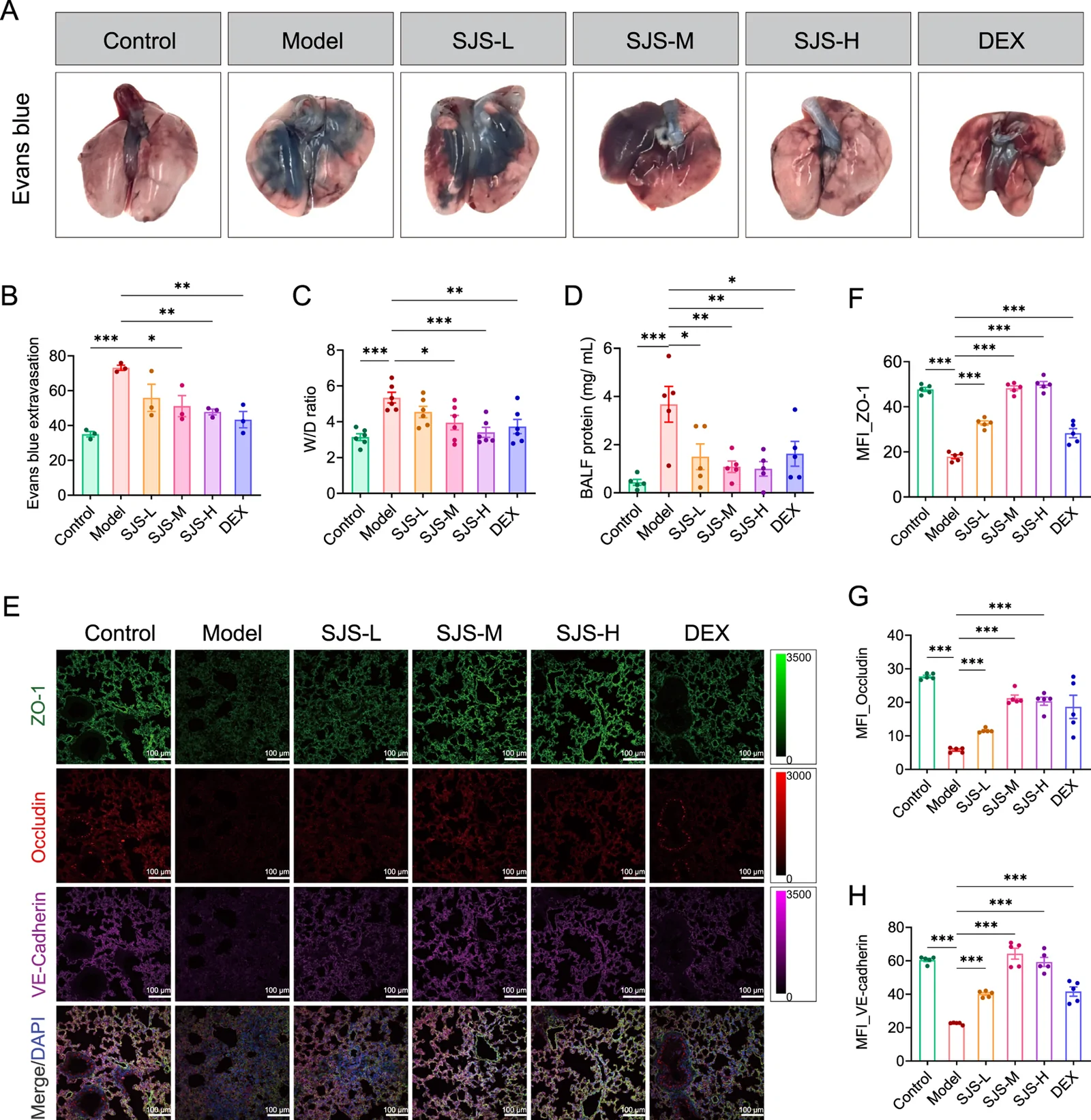
SJS preserves pulmonary barrier integrity after LPS challenge. **(A)** Representative gross images of Evans blue extravasation in lungs. **(B)** Quantification of Evans blue leakage. **(C)** Lung wet-to-dry (W/D) ratio. **(D)** Total protein concentration in BALF. **(E)** Representative multiplex immunofluorescence images of lung sections stained for ZO-1 (green), Occludin (red), VE-cadherin (magenta), and DAPI (blue). Scale bar = 100 μm. LUT scales shown at the right indicate fluorescence intensity in arbitrary units (a.u.). Identical LUT ranges were applied across all experimental groups within each fluorescence channel. **(F–H)** Quantification of ZO-1 **(F)**, Occludin **(G)**, and VE-cadherin **(H)** fluorescence intensity. Data are presented as mean ± SEM (B, n = 3; C, n = 6; D, F-H, n = 5). ^*^ *P* < 0.05, ^**^ *P* < 0.01, ^***^ *P* < 0.001 vs. Model.

### 3.3 Transcriptomic profiling identified a complement-associated neutrophil activation and NET formation program responsive to SJS

To characterize the transcriptional response to SJS pretreatment, RNA sequencing was performed on lung tissues from the Control, Model, and SJS groups (Figure 3A). Pearson correlation analysis showed high concordance among biological replicates (Supplementary Figure S2A), while principal component analysis revealed distinct clustering of the three groups (Figure 3B), indicating clear differences in their pulmonary transcriptional profiles.

**FIGURE 3:**
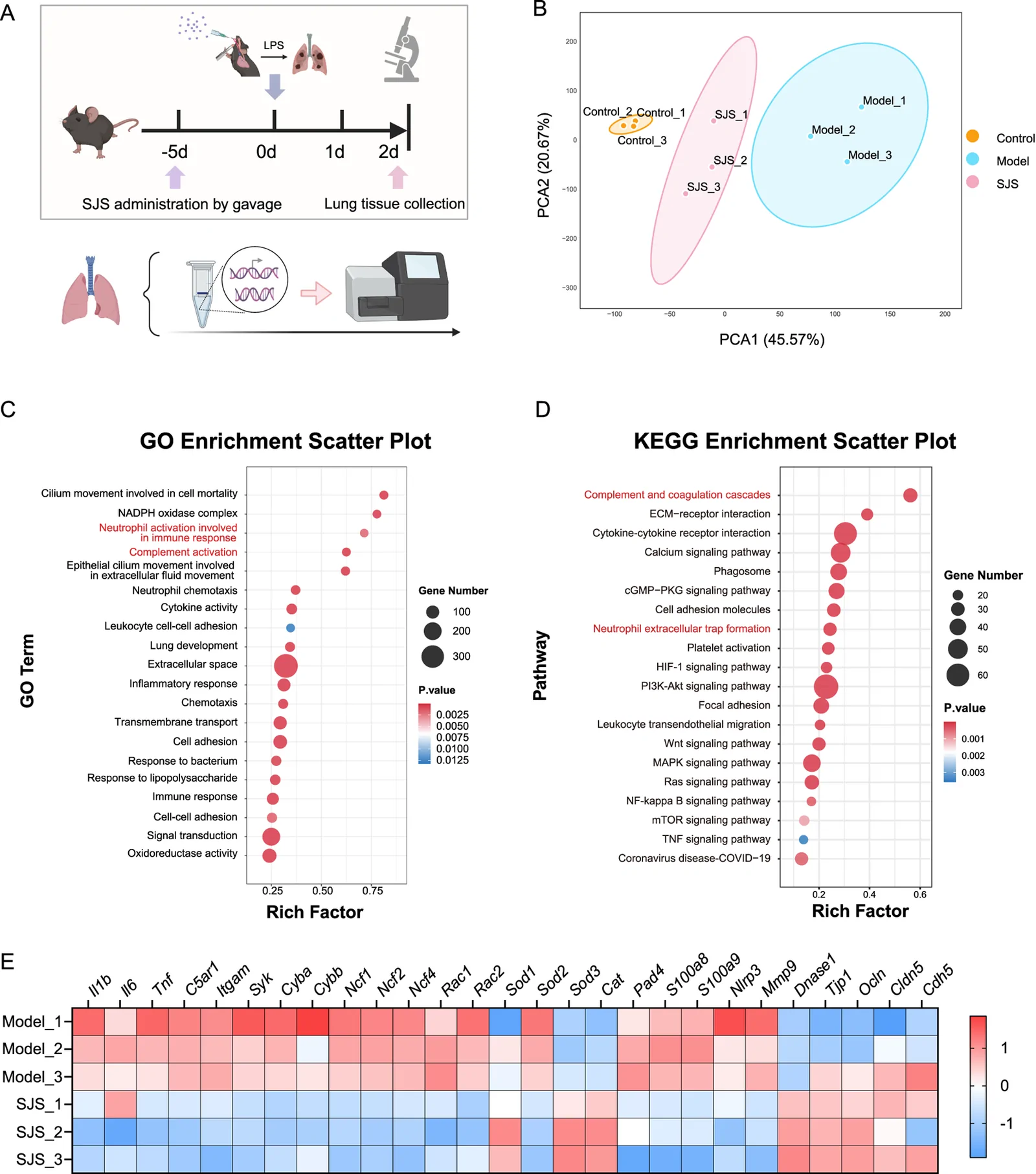
Transcriptomic profiling identifies complement activation and neutrophil extracellular trap formation as major pathways modulated by SJS in LPS-induced ALI. **(A)** Schematic illustration of the experimental design and lung tissue collection for transcriptomic analysis. **(B)** PCA showing sample clustering among the Control, Model, and SJS groups. **(C)** GO enrichment analysis of DEGs in the SJS vs. Model comparison. **(D)** KEGG pathway enrichment analysis of DEGs in the SJS vs. Model comparison. **(E)** Heatmap of 27 representative DEGs associated with inflammation, complement activation, NET formation, oxidative stress, and barrier related processes. Transcriptomic analysis was performed using lung tissues from three biologically independent mice per group (n = 3). Created in BioRender. ZHANG, D. (2026) https://BioRender.com/mj0banl

Differential expression analysis identified genes that were significantly altered between the SJS and Model groups. To identify genes dysregulated by LPS and regulated in the opposite direction by SJS, the differentially expressed genes from the Model *versus* Control and SJS *versus* Model comparisons were intersected. This analysis identified 2,175 genes, which were defined as SJS-responsive genes (Supplementary Figure S2B). GO enrichment analysis of these differentially expressed genes revealed significant enrichment of biological processes related to neutrophil activation, complement activation, and regulation of inflammatory responses (Figure 3C). KEGG enrichment analysis further identified the complement and coagulation cascades and neutrophil extracellular trap formation among the prominent pathways (Figure 3D). A heatmap of 27 representative differentially expressed genes selected according to their relevance to these pathways and the phenotypes examined in this study showed that genes associated with complement activation and NET formation, including *C5ar1* and *Padi4*, were increased in the Model group and reduced following SJS treatment (Figure 3E). Together, these transcriptomic findings indicated that SJS regulated pulmonary transcriptional programs involving complement activation, neutrophil activation, and NET formation.

### 3.4 SJS reduced pulmonary neutrophil accumulation and activation

Guided by the transcriptomic findings, neutrophil accumulation and activation in the lung were assessed by flow cytometry. LPS challenge increased the proportion of CD45^+^Ly6G^+^CD11b^+^ neutrophils in lung tissue, whereas SJS pretreatment reduced this increase, with the greatest reduction observed in the high-dose group (Figures 4A,B). The gating strategy used to identify pulmonary neutrophils is shown in Supplementary Figure S3. LPS challenge also increased CD11b mean fluorescence intensity on pulmonary neutrophils, which was reduced following SJS pretreatment (Figures 4C,D). Consistently, immunoblotting of whole-lung homogenates showed increased CD11b protein abundance in the Model group and lower levels after SJS pretreatment (Figures 4E,F). Immunohistochemical staining further revealed increased MPO and NE signals in lung tissue after LPS challenge, both of which were attenuated by SJS pretreatment (Figures 4G–I). These results show that SJS pretreatment reduced pulmonary neutrophil accumulation and attenuated neutrophil activation in LPS-induced ALI.

**FIGURE 4:**
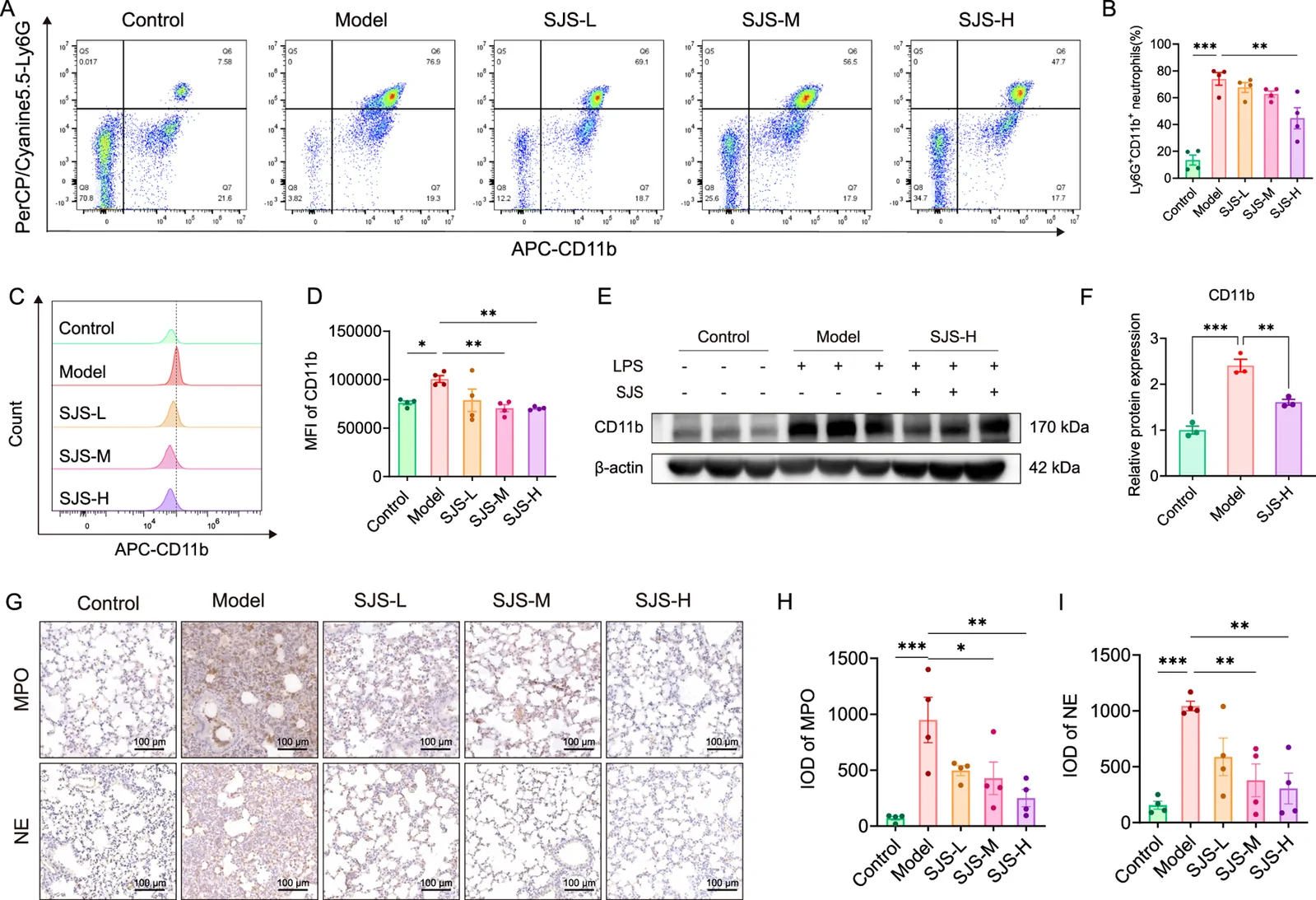
SJS reduced pulmonary neutrophil infiltration and activation after LPS challenge. **(A)** Representative flow cytometry plots showing pulmonary neutrophils gated as live CD45^+^Ly6G^+^CD11b^+^ cells. **(B)** Quantification of pulmonary CD45^+^Ly6G^+^CD11b^+^ neutrophils. **(C)** Representative histograms showing CD11b expression on pulmonary Ly6G^+^ neutrophils. **(D)** Quantification of CD11b MFI. **(E)** Representative immunoblots of CD11b in lung homogenates from the Control, Model, and SJS-H groups. **(F)** Densitometric analysis of CD11b expression. **(G)** Representative IHC images of MPO and NE in lung tissue. Scale bar, 100 μm. **(H,I)** Quantification of IOD values for MPO and NE. Data are presented as mean ± SEM (B, D, H, I, n = 4; F, n = 3). ^*^ *P* < 0.05, ^**^ *P* < 0.01, ^***^ *P* < 0.001 vs. Model.

### 3.5 SJS reduced pulmonary NET formation

We next examined whether the reduction in pulmonary neutrophil accumulation and activation was accompanied by decreased NET formation. Immunofluorescence staining showed increased colocalization of MPO and Cit-H3 in the lungs of Model mice, whereas these signals were attenuated by SJS pretreatment. Quantitative analysis confirmed reductions in MPO and Cit-H3 fluorescence intensities and the MPO/Cit-H3 colocalized area in the SJS-treated groups (Figures 5A–D). Consistent with these findings, MPO-DNA complexes in lung homogenates were increased after LPS challenge, reaching 70.98 ng/mL in the Model group, and were reduced to 38.07 ng/mL in the high-dose SJS group (Figure 5E). Immunoblotting further showed that LPS challenge increased PAD4 and Cit-H3 protein abundance, whereas SJS pretreatment attenuated these increases (Figures 5F–H). Together, these results show that SJS pretreatment reduced pulmonary NET formation in LPS-induced ALI.

**FIGURE 5:**
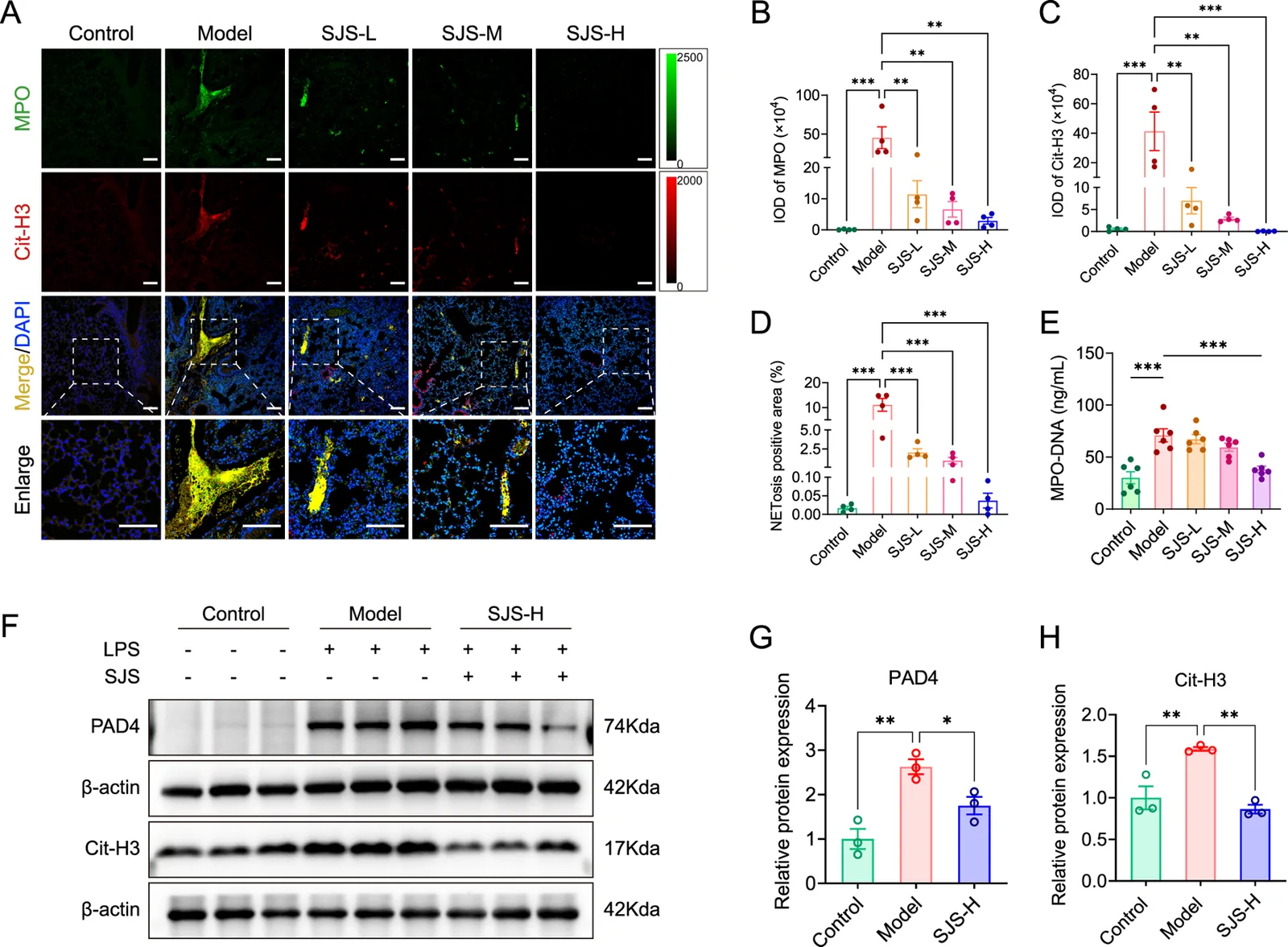
SJS suppressed pulmonary NET formation after LPS challenge. **(A)** Representative immunofluorescence images of MPO (green) and Cit-H3 (red) in lung sections. Nuclei were counterstained with DAPI (blue). Yellow signal indicates colocalization. Scale bar = 100 μm. LUT scales indicate fluorescence intensity in arbitrary units (a.u.), with identical display ranges applied across groups within each fluorescence channel. **(B,C)** Quantification of IOD values for MPO and Cit-H3. **(D)** Quantification of NET-positive area. **(E)** MPO-DNA complexes in lung homogenates. **(F)** Immunoblot analysis of PAD4 and Cit-H3 in lung tissue from the Control, Model, and SJS-H groups. **(G,H)** Relative expression of PAD4 and Cit-H3. Data are presented as mean ± SEM (A, n = 6; B-D, n = 5; E, n = 6; G, H, n = 3). ^*^ *P* < 0.05, ^**^ *P* < 0.01, ^***^ *P* < 0.001 vs. Model.

### 3.6 SJS suppressed C5a/C5aR1 associated neutrophil activation and NET formation

Given the enrichment of complement related pathways in the transcriptomic analysis, C5a levels were measured in plasma and lung homogenates. LPS challenge increased C5a levels from 120.1 to 259.4 pg/mL in plasma and from 78.9 to 175.7 pg/mL in lung homogenates. SJS pretreatment reduced plasma C5a levels to 156.2, 153.1, and 122.8 pg/mL and C5a levels in lung homogenates to 141.3, 115.7, and 102.9 pg/mL in the low, medium, and high dose groups, respectively (Figures 6A,B). Consistently, *C5ar1* mRNA expression was increased in the lungs of model mice and reduced following SJS pretreatment (Figure 6C). Immunohistochemical staining further showed increased C5aR1 expression in the lung after LPS challenge, which was attenuated by SJS pretreatment, as confirmed by quantitative analysis (Figures 6D,E). Thus, LPS challenge increased circulating and pulmonary C5a levels and enhanced C5aR1 expression in the lung, whereas SJS pretreatment attenuated these changes. Reanalysis of the GSE264032 single-cell RNA-sequencing dataset showed that *C5ar1* transcripts were most frequently detected in neutrophils (78.9%), followed by macrophages (60.0%) and monocytes (23.2%) (Supplementary Figure S4). Primary neutrophils with a purity greater than 90% were used for subsequent experiments (Supplementary Figure S5). CCK-8 analysis showed that 10% SJS containing serum did not reduce neutrophil viability, whereas 20% SJS containing serum decreased cell viability. Therefore, 10% SJS containing serum was used in the following experiments (Supplementary Figure S6A). In the Transwell assay, C5a increased the numbers of neutrophils migrating to the lower surface of the membrane and into the lower chamber. Both responses were reduced by SJS containing serum and PMX205 (Figures 6F–I). Neither SJS containing serum nor PMX205 altered neutrophil apoptosis under the same experimental conditions (Supplementary Figure S6B,C). C5a stimulation also increased MPO and Cit-H3 fluorescence and expanded the NET-positive area, whereas these changes were attenuated by SJS containing serum and PMX205 (Figures 6J–M). Flow cytometric analysis further showed that C5a increased intracellular Ca^2+^ and ROS levels in neutrophils, both of which were reduced by SJS containing serum and PMX205 (Figures 6N–Q). Collectively, SJS pretreatment reduced C5a levels and pulmonary C5aR1 expression in LPS-induced ALI, while SJS containing serum attenuated C5a-induced neutrophil migration, intracellular Ca^2+^ and ROS responses, and NET formation *in vitro*.

**FIGURE 6:**
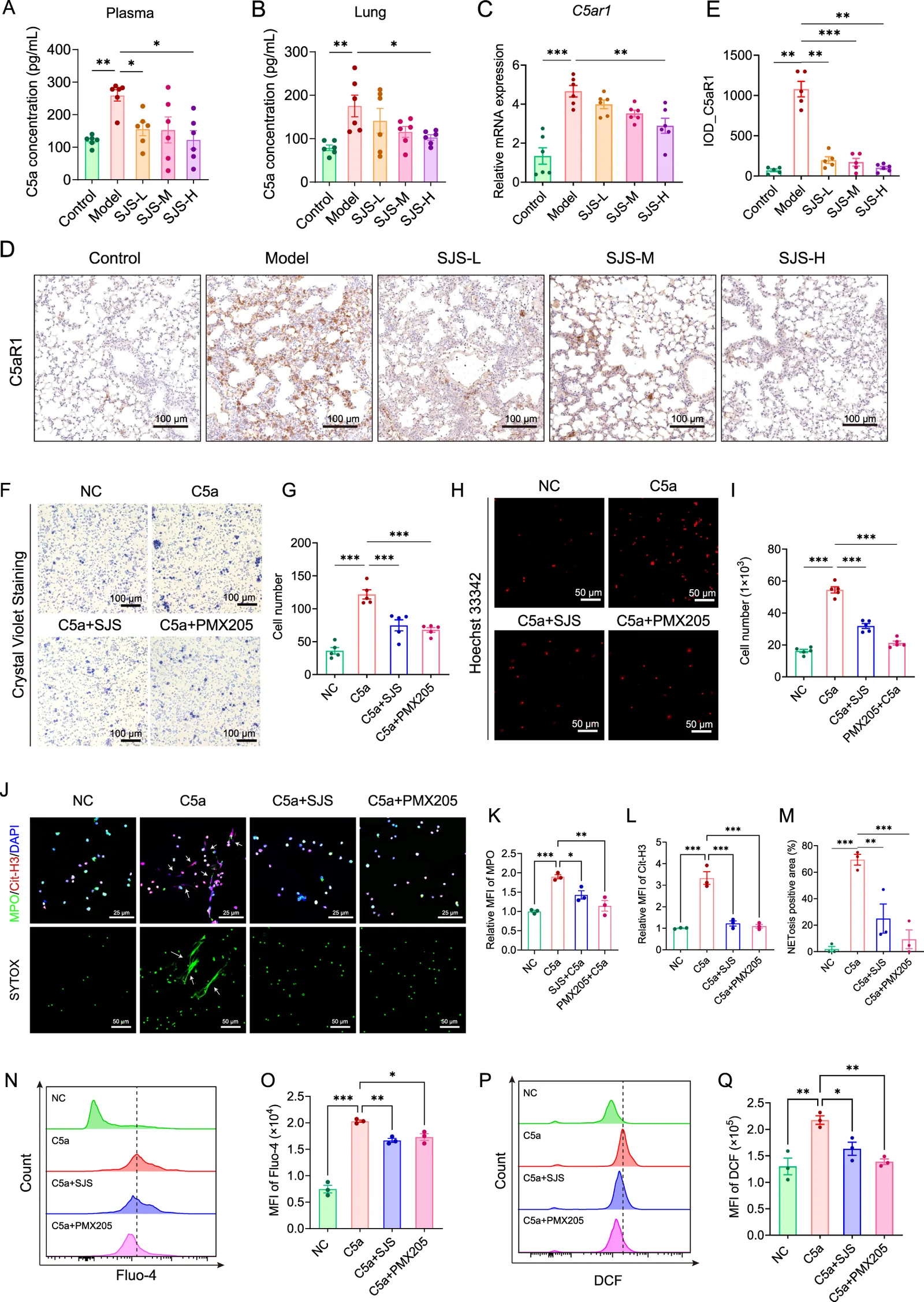
SJS reduced C5a levels and C5aR1 expression, while SJS containing serum suppressed C5a-induced neutrophil responses. **(A,B)** C5a concentrations in plasma and lung homogenates. **(C–E)** Lung *C5ar1* mRNA expression, representative C5aR1 immunohistochemistry, and IOD quantification. Scale bar = 100 μm. **(F–I)** Transwell migration assessed by crystal violet and Hoechst 33342 staining, with corresponding quantification. Hoechst 33342 fluorescence is pseudocolored red. Scale bars = 100 μm **(F)** and 50 μm **(H)**. **(J–M)** MPO/Cit-H3 immunofluorescence, SYTOX Green staining, relative MFI of MPO and Cit-H3, and NET-positive area. Scale bars = 20 μm. **(N–Q)** Flow cytometric analysis of intracellular Ca^2+^ and ROS using Fluo-4 AM and DCFH-DA, respectively. Data are presented as mean ± SEM (A-C, *n* = 6; E and G, *n* = 5; I, *n* = 4; K-M, O, and Q, *n* = 3). ^*^ *P* < 0.05, ^**^ *P* < 0.01, and ^***^ *P* < 0.001.

### 3.7 SJS additionally suppressed PMA-induced NET formation

To determine whether the inhibitory effect of SJS-containing serum extended beyond C5a stimulation, primary neutrophils were treated with PMA. PMA increased the fluorescence intensities of MPO, NE, PAD4, and Cit-H3, whereas SJS-containing serum attenuated these increases without affecting their basal levels in unstimulated neutrophils (Figures 7A–E). PMA induced extensive extracellular web-like structures together with increased extracellular DNA and Cit-H3 signals, as shown by SYTOX Green staining, MPO/Cit-H3 immunofluorescence, and scanning electron microscopy (Figure 7F). Both SJS-containing serum and GSK484 attenuated PMA-induced NET formation, as evidenced by fewer extracellular web-like structures and lower relative Cit-H3 MFI and NET-positive area (Figures 7F–H). The inhibitory effect of SJS-containing serum on NET formation was therefore not limited to C5a stimulation.

**FIGURE 7:**
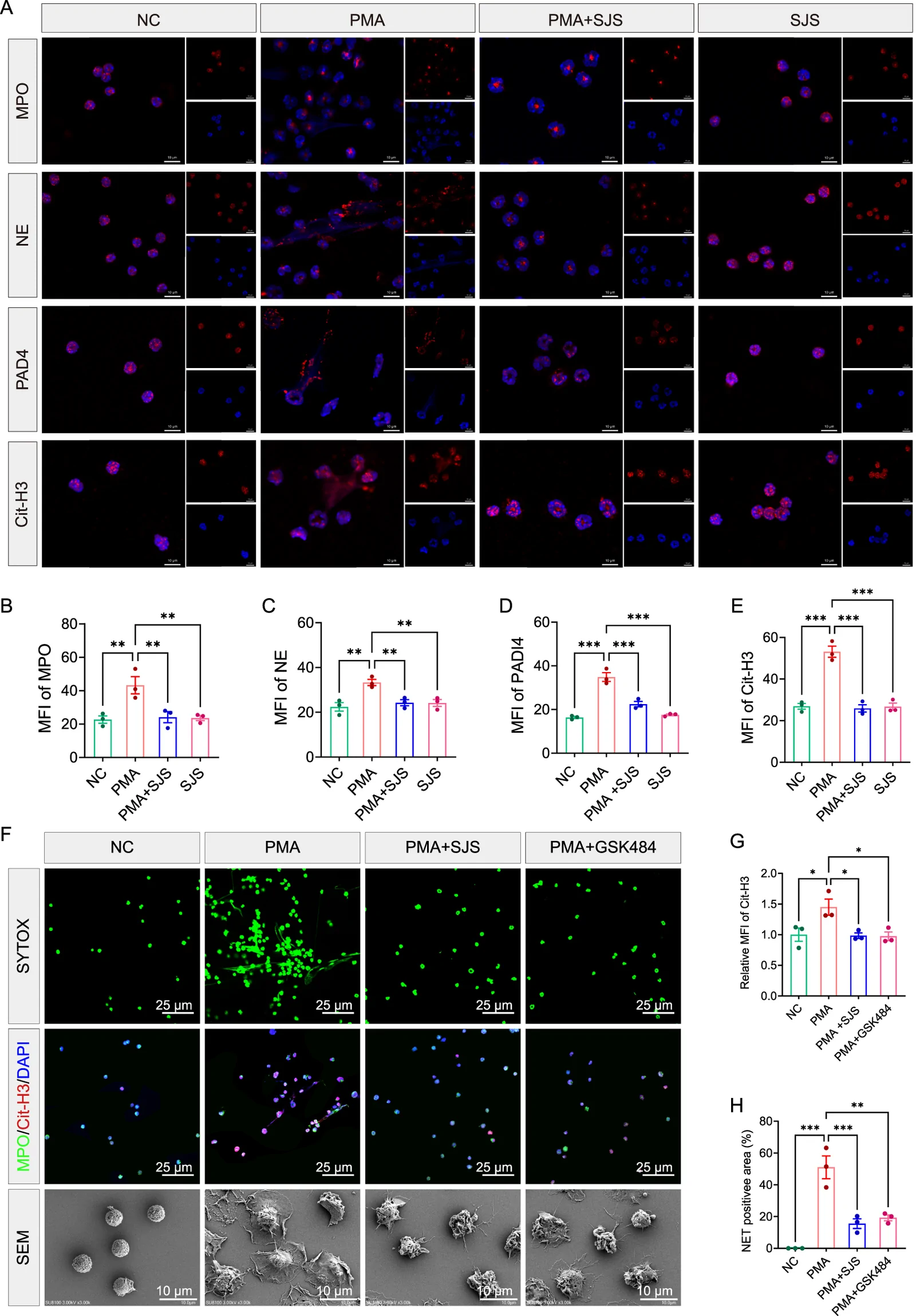
SJS additionally suppressed PMA-induced NET formation *in vitro*. **(A)** Representative immunofluorescence images of MPO, NE, PAD4, and Cit-H3 in the NC, PMA, PMA + SJS, and SJS groups. Target proteins, red; DAPI, blue. Insets show individual fluorescence channels. **(B–E)** MFI of MPO **(B)**, NE **(C)**, PAD4 **(D)**, and Cit-H3 **(E)**. **(F)** Representative SYTOX Green staining, MPO/Cit-H3 immunofluorescence, and scanning electron microscopy images of neutrophils in the NC, PMA, PMA + SJS, and PMA + GSK484 groups. MPO, green; Cit-H3, red; DAPI, blue. Scale bars are as indicated. **(G,H)** Relative Cit-H3 MFI **(G)** and NET-positive area **(H)**. Data are presented as mean ± SEM (n = 3). ^*^ *P* < 0.05, ^**^ *P* < 0.01, and ^***^ *P* < 0.001 vs. PMA.

### 3.8 Chemical profiling characterized the constituents and circulating components of SJS

The chemical profile of the lyophilized aqueous extract of SJS was characterized by UHPLC-Q-Orbitrap HRMS, with total ion chromatograms obtained in positive and negative ion modes (Figures 8A,B). A total of 95 constituents were identified or tentatively characterized and classified into 11 chemical classes (Supplementary Table S4). The major chemical classes were anthraquinones and anthrones, phenylpropanoids and tannins, and terpenoids, which accounted for 28 (29.47%), 18 (18.95%), and 13 (13.68%) constituents, respectively (Figure 8C).

**FIGURE 8:**
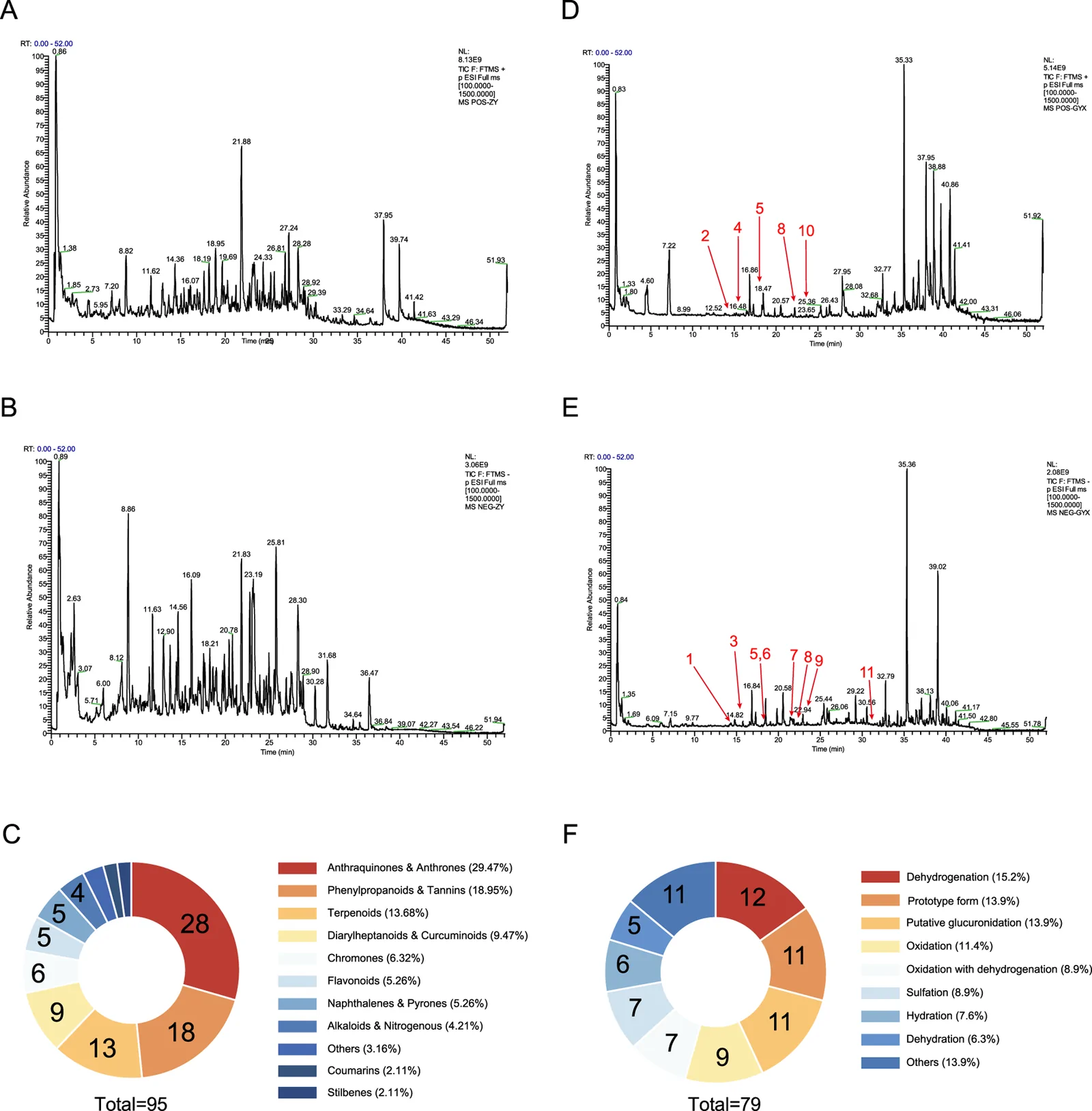
Chemical profile of SJS and SJS-derived chemical entities detected in serum. **(A,B)** Total ion chromatograms of the SJS extract in positive and negative ionization modes, respectively. **(C)** Chemical class distribution of the 95 constituents characterized in SJS. **(D,E)** Total ion chromatograms of serum from SJS-administered rats. Red arrows indicate the 11 prototype constituents identified by comparison with serum from untreated control rats. **(F)** Distribution of the 79 serum-detected entries, including 11 prototype constituents and 68 putative metabolites.

To characterize SJS-derived chemical entities detected in the circulation, serum profiles from untreated control rats and SJS-administered rats were compared. A total of 80 SJS-related ion signals were detected in positive and negative ionization modes (Figures 8D,E). After merging entries assigned to the same compound but detected as different adduct ions, 79 unique serum-detected entries were retained, comprising 11 prototype constituents and 68 putative metabolites (Supplementary Table S5). The prototype constituents included ferulic acid, isololiolide, danthron, kaempferol, emodin, and laccaic acid D, with the complete list provided in Supplementary Table S5. The putative metabolites were primarily assigned to dehydrogenation, glucuronidation, oxidation, combined oxidation and dehydrogenation, sulfation, hydration, dehydration, and other biotransformation pathways (Figure 8F). These analyses characterized the chemical composition of the SJS extract and the SJS-derived chemical entities detected in serum, providing a candidate pool for the subsequent evaluation of circulating active constituents.

### 3.9 Ferulic acid was identified as a candidate circulating constituent of SJS and attenuated C5a-induced NET formation *in vitro*

Ferulic acid was detected among the prototype constituents in SJS-containing serum (Figure 9A). Following intragastric administration of SJS, its plasma concentration reached 10.3 μg/mL at 0.5 h and gradually declined to approximately 1.6 μg/mL at 4 h (Figure 9B). BLI analysis showed concentration-dependent binding between ferulic acid and recombinant human C5a, with an apparent *K*_D_ value of 21.65 μM (Figures 9C,D). Molecular docking predicted a possible binding mode of ferulic acid with C5a, involving Arg27, Arg62, and Lys68 (Figures 9E,F). In primary neutrophils, ferulic acid attenuated the C5a-induced increases in Cit-H3 fluorescence and NET-positive area (Figures 9G–I). These results identify ferulic acid as a circulating constituent of SJS with *in vitro* activity against C5a-induced NET formation.

**FIGURE 9:**
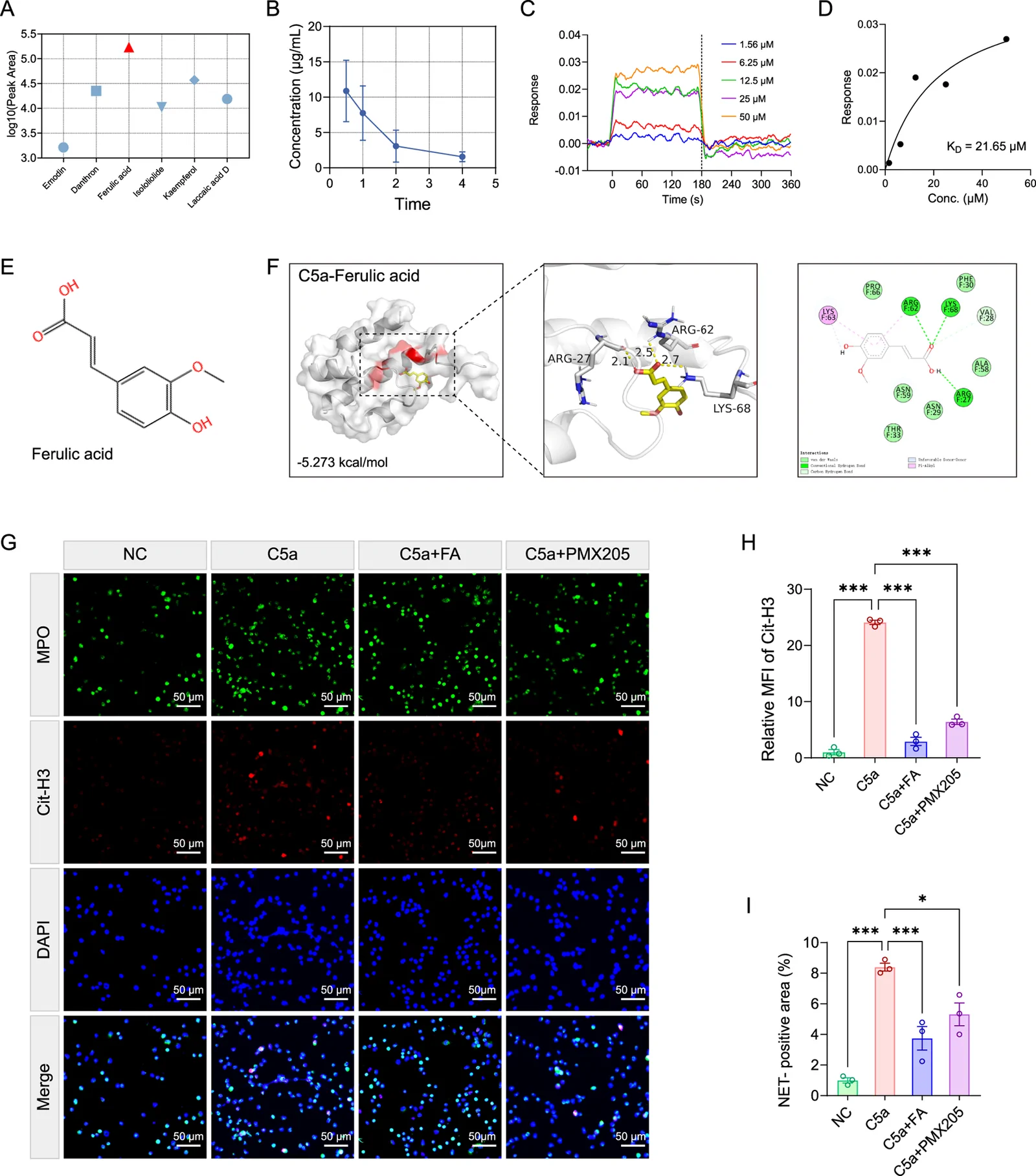
Identification and functional evaluation of ferulic acid as a candidate circulating constituent of SJS. **(A)** Peak areas of selected prototype constituents detected in serum after SJS administration. The red triangle indicates ferulic acid. **(B)** Plasma concentration-time profile of ferulic acid after SJS administration. **(C,D)** Representative BLI binding curves and steady-state affinity analysis of ferulic acid binding to recombinant human C5a. **(E)** Chemical structure of ferulic acid. **(F)** Predicted binding mode of ferulic acid with C5a, showing interactions with Arg27, Arg62, and Lys68. **(G)** Representative MPO/Cit-H3 immunofluorescence images of primary neutrophils in the indicated groups. MPO, green; Cit-H3, red; DAPI, blue. Scale bar = 50 μm. **(H,I)** Relative Cit-H3 MFI and NET-positive area. Data are presented as mean ± SEM (B, n = 6; H, I, n = 3). ^*^ *P* < 0.05, ^**^ *P* < 0.01 and ^***^ *P* < 0.001 for the indicated comparisons.

## 4 Discussion

In the present study, SJS pretreatment attenuated LPS-induced ALI, accompanied by reduced pulmonary neutrophil accumulation and NET formation and better preservation of alveolar-capillary barrier integrity. Transcriptomic profiling identified complement activation, neutrophil activation, and NET formation as prominent SJS-responsive processes, and subsequent animal and primary neutrophil experiments supported the involvement of C5a/C5aR1-associated neutrophil responses. Ferulic acid was further identified as a candidate circulating constituent that attenuated C5a-induced NET formation *in vitro*. These findings identify C5a/C5aR1-associated neutrophil activation and excessive NET formation as important SJS-responsive processes in ALI (Figure 10).

**FIGURE 10:**
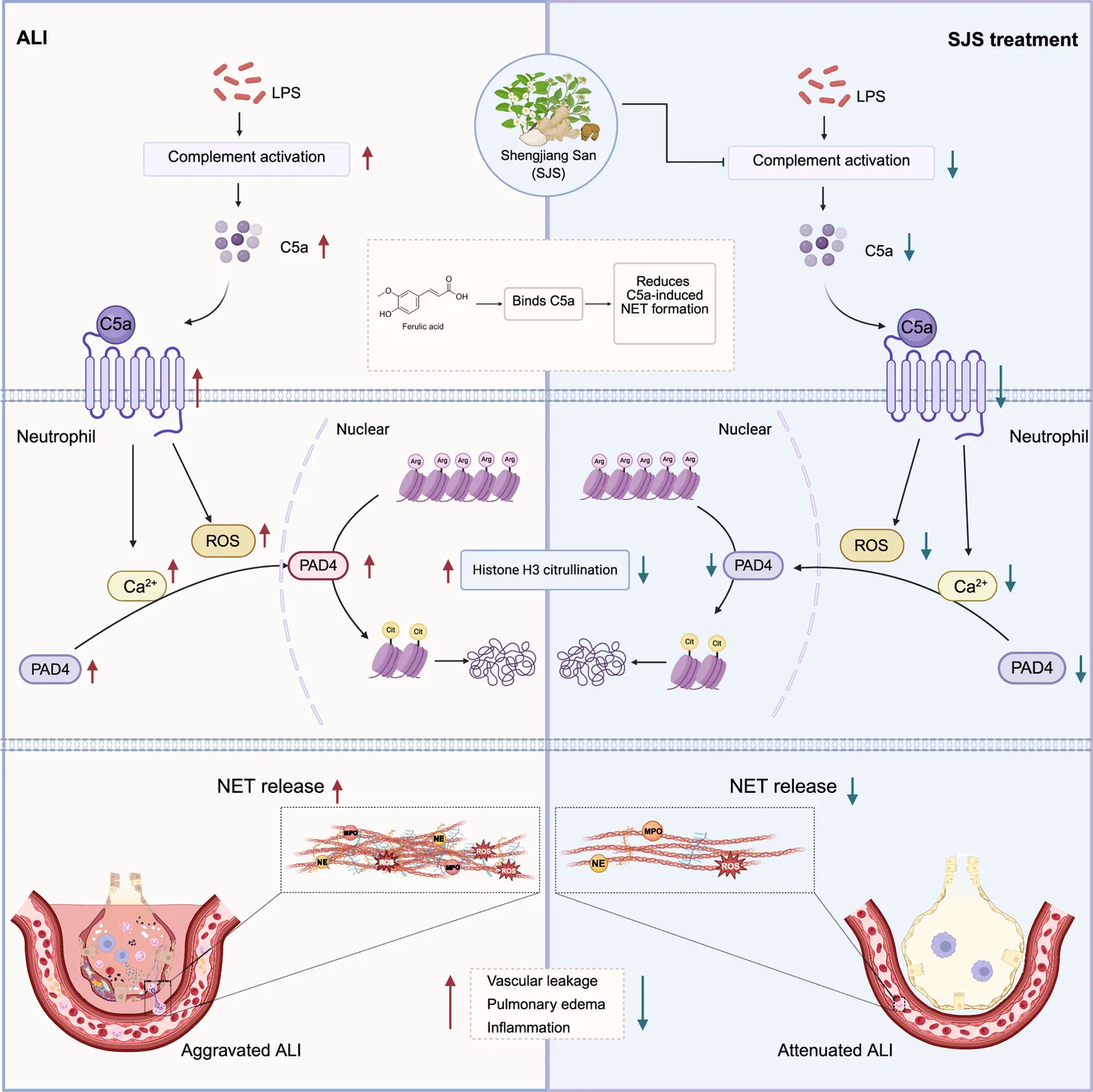
SJS suppresses C5a/C5aR1 associated NET formation to alleviate acute lung injury. Created in BioRender. ZHANG, D. (2026) https://BioRender.com/n147ips

ALI and ARDS are biologically heterogeneous syndromes characterized by alveolar epithelial and endothelial injury, dysregulated innate immune responses, coagulation abnormalities, and impaired tissue repair (Matthay et al., 2019). The contribution of these processes varies across etiologies and disease stages (Bos and Ware, 2022; Matthay et al., 2019), and secondary analyses of randomized trials have shown that ARDS subphenotypes may respond differently to the same intervention (Calfee et al., 2014; Famous et al., 2017; Calfee et al., 2018). This heterogeneity highlights the importance of targeting defined pathogenic processes. Previous mechanistic studies of SJS in sepsis-induced lung injury focused on macrophage pyroptosis involving NF-κB/NLRP3 signaling (Yan et al., 2023). The present findings extend this work by showing that SJS also attenuated neutrophil activation and excessive NET formation associated with C5a/C5aR1 signaling in LPS-induced ALI.

Among the upstream pathways that regulate neutrophil activation, C5a/C5aR1 signaling is particularly relevant to inflammatory lung injury. Under inflammatory conditions, this pathway can promote NET formation, and inhibition of C5aR1 has been shown to reduce NET-associated lung inflammation and tissue injury *in vivo* (Silva et al., 2023). Single-cell analysis further provided cellular context for this pathway, with *C5ar1* transcripts most frequently detected in neutrophils, while substantial detection was also observed in macrophages and monocytes. In the LPS-induced ALI model, SJS pretreatment reduced the elevations of C5a in both plasma and lung homogenates and attenuated pulmonary C5aR1 upregulation. In primary neutrophils, SJS-containing serum suppressed C5a-induced migration, intracellular Ca^2+^ mobilization, ROS generation, and NET formation, while PMX205 produced a similar inhibitory pattern. The concordant *in vivo* and *in vitro* findings support the involvement of C5a/C5aR1-associated neutrophil activation in the effects of SJS but do not establish that SJS directly antagonizes C5aR1.

These C5a/C5aR1-associated responses are pathologically relevant because neutrophils, although essential for host defense, can aggravate pulmonary inflammation and tissue injury when their accumulation and activation become excessive or sustained (Zhang et al., 2024). Neutrophil function is context dependent, as distinct activation states may either amplify tissue injury or promote resolution (Wang et al., 2022; Tavares et al., 2025). In ARDS, pulmonary neutrophil accumulation has been associated with impaired gas exchange and increased lung protein permeability, whereas sustained alveolar neutrophilic inflammation has been associated with greater disease severity and mortality (Steinberg et al., 1994; Weiland et al., 1986). In the present model, SJS pretreatment reduced the proportion of CD45^+^Ly6G^+^CD11b^+^ neutrophils in lung tissue and lowered surface CD11b expression on these cells. Reduced CD11b protein abundance and MPO and NE immunoreactivity in lung tissue provided additional evidence that SJS attenuated pulmonary neutrophil accumulation and activation. These findings indicate that SJS restrained pulmonary neutrophil accumulation and activation, consistent with its effects on C5a/C5aR1-associated neutrophil responses.

NET formation is an antimicrobial effector response of activated neutrophils (Brinkmann et al., 2004), but excessive NET accumulation has been associated with greater disease severity and mortality in ARDS (Lefrançais et al., 2018). In experimental ALI, excessive NET accumulation can disrupt the alveolar-capillary barrier by inducing alveolar epithelial necroptosis (Sha et al., 2024) and damaging the pulmonary microvascular endothelium (Caudrillier et al., 2012). NETs can also amplify pulmonary inflammation by promoting alveolar macrophage pyroptosis (Li et al., 2021) and contribute to immunothrombosis through STING-dependent tissue factor expression in endothelial cells (Zhu et al., 2023). Because no individual marker is specific for NETs, complementary imaging and biochemical approaches were used to assess NET formation (Boeltz et al., 2019). SJS pretreatment reduced pulmonary MPO/Cit-H3 colocalization and MPO-DNA complexes and lowered PAD4 and Cit-H3 protein abundance. In primary neutrophils, SJS-containing serum attenuated NET formation induced by both C5a and PMA. Because PMA bypasses cell surface receptor signaling and induces NET formation through PKC activation and ROS production by NADPH oxidase (Gray et al., 2013), the PMA findings indicate that the inhibitory effect of SJS-containing serum was not restricted to C5a stimulation. *In vivo*, reduced NET formation coincided with less vascular leakage and pulmonary edema and better preservation of ZO-1, Occludin, and VE-cadherin. These findings support an association between reduced NET formation and preservation of alveolar-capillary barrier integrity, although the broader anti-inflammatory effects of SJS may also have contributed to the improvement in barrier function.

Among the prototype constituents detected in serum after SJS administration, ferulic acid showed a relatively prominent signal in the serum mass spectrometric profile and measurable plasma exposure. It was therefore selected as a candidate circulating constituent for further characterization. BLI showed that the binding of ferulic acid to C5a increased with concentration, yielding an apparent K_D_ of 21.65 μM. The peak plasma concentration of ferulic acid was approximately 53 μM, which was in the same micromolar range as the apparent *K*_*D*_. However, this comparison does not establish target engagement *in vivo*. Molecular docking predicted a possible binding pose involving Arg27, Arg62, and Lys68 of C5a. Structural studies have shown that C5a binds to C5aR1 through multiple contact regions, with Arg27 contributing to an extracellular recognition interface and Lys68 located within the C-terminal segment that enters the receptor binding cavity (Feng et al., 2023; Yang et al., 2024). In primary neutrophils, ferulic acid reduced the increases in Cit-H3 fluorescence and NET area following C5a stimulation. The combined pharmacochemical, biophysical, structural, and cellular evidence supports ferulic acid as a candidate circulating constituent of SJS with activity against C5a-induced NET formation *in vitro*.

## 5 Limitations

Several limitations should be considered. First, the present study evaluated SJS in an LPS-induced mouse model of ALI using a pretreatment regimen. Although this model captures key features of inflammation-driven lung injury, ALI/ARDS has diverse etiologies and disease stages, and direct clinical evidence for classical SJS in this setting remains limited. Additional models, treatment initiated after injury onset, and prospective clinical evaluation are therefore needed to determine whether the protective effects observed here can be translated beyond the experimental setting. Mechanistically, this study focused on neutrophil activation and NET formation associated with C5a/C5aR1 signaling. However, SJS-containing serum also inhibited PMA-induced NET formation, indicating that its effects may involve additional NET-inducing pathways or shared downstream signals that were not explored here. In addition, although ferulic acid was identified as a candidate circulating constituent with activity against C5a-induced NET formation, its contribution to the overall *in vivo* efficacy of SJS remains unclear, and other circulating constituents were not systematically evaluated. Finally, generative AI was used only for language refinement and not for study design, data analysis, or interpretation. All AI-assisted text was reviewed and verified by the authors.

## 6 Conclusion

SJS pretreatment attenuated ALI caused by LPS, accompanied by reduced pulmonary neutrophil accumulation and NET formation and preservation of alveolar capillary barrier integrity. Transcriptomic analysis, animal experiments, and studies in primary neutrophils consistently linked C5a/C5aR1 signaling to the neutrophil activation and NET formation attenuated by SJS. Serum pharmacochemistry and subsequent *in vitro* characterization further supported ferulic acid as a candidate circulating constituent of SJS that reduced NET formation following C5a stimulation. These findings provide a rationale for further evaluation of SJS in ALI, particularly for its potential to restrain neutrophil responses associated with complement activation and excessive NET formation.

## Data Availability

The RNA-sequencing data generated in this study are deposited in the NCBI Gene Expression Omnibus (GEO) under accession number GSE345908. The publicly available single-cell RNA-sequencing dataset reanalyzed in this study is available in the NCBI Gene Expression Omnibus (GEO) under accession number GSE264032. Additional data supporting the findings of this study are included in the article and its Supplementary Material.
